# p38 Regulates FoxO3a-Mediated SOD2 Expression to Prevent Cd-Induced Oxidative Stress in Neuronal Cells

**DOI:** 10.3390/ijms262210919

**Published:** 2025-11-12

**Authors:** Tianji Lin, Shijuan Ruan, Xinyu Liu, Fangfei Li, Hangqian Zhang, Fei Zou, Bin Wang

**Affiliations:** 1 Department of Occupational Health and Occupational Medicine, School of Public Health, Southern Medical University, Guangzhou 510515, China; lintianji@126.com (T.L.); ruansj2025@163.com (S.R.); 13060604992@163.com (X.L.); 13172396244@163.com (F.L.); 15257956196@163.com (H.Z.); 2 Guangzhou Center for Disease Control and Prevention (Guangzhou Health Supervision Institute), Guangzhou 511400, China; 3 Sichuan Center for Disease Control and Prevention, Chengdu 610041, China

**Keywords:** cadmium, p38, FoxO3a, SOD2, oxidative stress

## Abstract

Cadmium (Cd), an environmental toxin, may cause neurological disorders. We studied the role and activation mechanism of FoxO3a in Cd-induced oxidative stress. In addition to oxidative stress, Cd activated the antioxidant defense system in neuronal cells. Furthermore, by using Western blot and confocal microscopy, we found that Cd induced nuclear expression of FoxO3a. Importantly, knockdown of FoxO3a significantly suppressed its target SOD2 protein expression and elevated the level of intracellular ROS, ultimately reducing cell viability in Cd-exposed neuronal cells. These results suggest the protective effect of FoxO3a is associated with oxidative stress resistance. Then, we investigated the activation mechanism of FoxO3a. Our results indicate that the nuclear expression of FoxO3a by Cd may be independent of Akt, which is generally regarded as an important negative regulator of FoxO3a. Furthermore, we found that p38 regulated the nuclear expression of FoxO3a in Cd-exposed cells. Finally, we demonstrate that the p38-FoxO3a pathway inhibits Cd-induced oxidative stress. These signaling molecules may be used as a novel biological marker of Cd-induced oxidative stress and provide potential therapeutic approaches for it.

## 1. Introduction

Cadmium (Cd) is a heavy metal that has received considerable concern environmentally and occupationally. Several studies have shown that not only certain occupations and tobacco smoke but also Cd exposure in the diet and environment are important sources of Cd exposure, which may cause health problems [1,2,3]. Due to the chronic exposure, combined with the long half-life of Cd in the human body [4,5], Cd can increase the blood–brain barrier permeability and accumulate in the brain, contributing to nervous system damage [6,7].

Increasing evidence suggests that oxidative stress contributes to Cd-induced cytotoxicity [8,9,10]. Meanwhile, the antioxidant defense system could be triggered to mitigate oxidative damage. SOD1 is a constitutively expressed antioxidase, while manganese superoxide dismutase (SOD2) can be induced by different contexts [11,12]. It is well established that SOD2 scavenges and accelerates the dismutation of superoxide into molecular oxygen and water. The level of SOD2 plays an important role in maintaining the function of the antioxidant system, which can promote cell adaptation and survival [13,14].

FoxO3a (also known as FKHR-L1) is a member of the FoxO subfamily of forkhead transcription factors, which regulates a wide range of biological processes, including apoptosis, metabolism, proliferation, and oxidative stress resistance [15,16]. Several studies have reported that FoxO3a mediates cell death through up-regulating apoptotic gene expression [17,18]. However, FoxO3a has also been found to serve a protective role through inducing pro-survival molecules (e.g., SOD2, ATM) in vivo and in vitro [19,20,21]. What role FoxO3a plays in Cd-induced oxidative stress remains unclear.

The nuclear expression and transactivation activity of FoxO3a are regulated by post-translational modifications, such as phosphorylation, acetylation, ubiquitination, and methylation [22,23,24]. Phosphorylation is the most common form of modification. FoxO3a is an important downstream target of the PI3K/Akt signaling pathway. Akt-mediated phosphorylation of FoxO3a results in its export into the cytoplasm and loss of transcriptional activity [25]. The stress-activated mitogen-activated protein kinases (MAPKs) include ERK (extracellular signal-regulated kinase), p38, and JNK (c-Jun NH2-terminal kinase), which have also been reported to regulate the nuclear expression of FoxO3a [26,27]. However, the upstream regulator of FoxO3a in Cd-induced oxidative stress is not understood.

In this study, we demonstrate that FoxO3a protects neuronal cells against Cd-induced oxidative stress by promoting SOD2 activity and that FoxO3a is regulated by p38.

## 2. Results

### 2.1. Cd Induces Oxidative Stress and Activates Antioxidant Defense Response in Neuronal Cells

**Figure 1:**
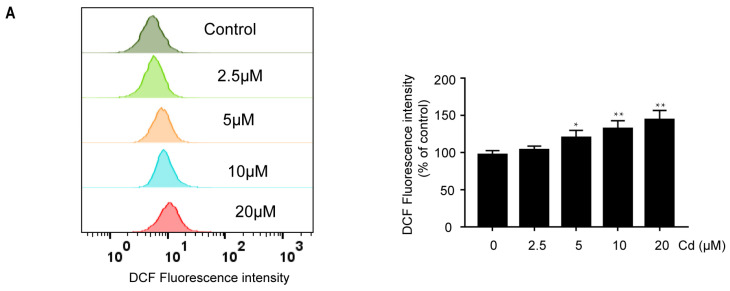

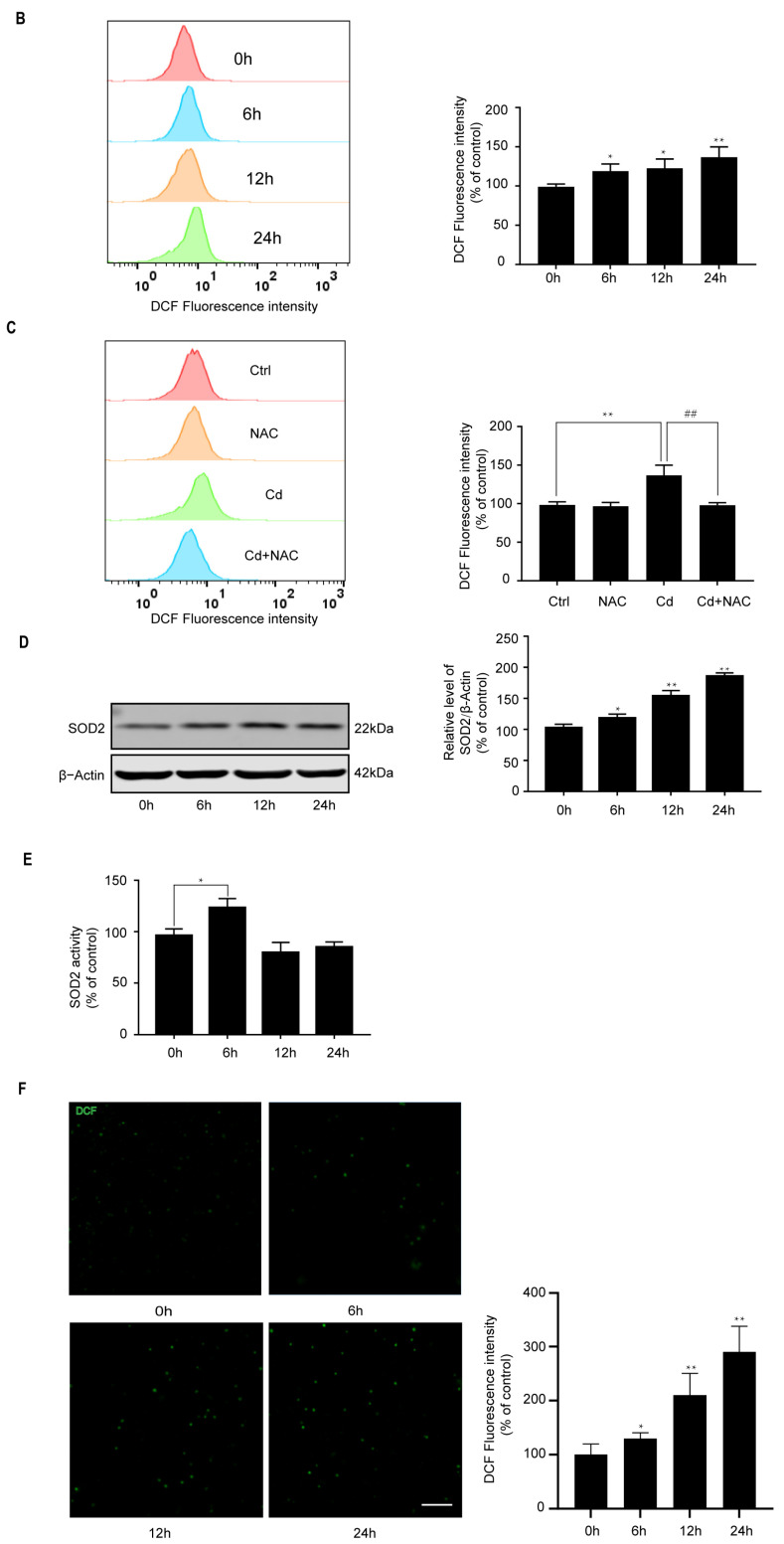
Cd induced oxidative stress in neuronal cells. (**A**) SH-SY5Y cells were treated with indicated concentrations of Cd for 24 h, and subsequently, ROS levels were analyzed by flow cytometry. (**B**) SH-SY5Y cells were incubated with 5 μM Cd for various time periods. The formation of ROS was measured by flow cytometry. (**C**) SH-SY5Y cells were exposed to 5 μM Cd and 2.5 mM NAC alone or together for 24 h; subsequently, ROS levels were analyzed by flow cytometry. (**D**,**E**) SH-SY5Y cells were exposed to 5 μM Cd at the indicated times. Western Blot analysis was performed to determine SOD2 protein expression, and a CuZn/Mn-SOD Assay Kit was used to measure SOD2 activity. (**F**) Rat cerebral cortical neurons were incubated with 5 μM Cd for various time periods, and subsequently, ROS levels were analyzed by fluorescence microscopy. All results are representative of three independent experiments. The provided scale bar in the merged image represents 200 μm. * *p* < 0.05, ** *p* < 0.01 versus the control group; ## *p* < 0.01 versus the Cd group.

We used SH-SY5Y cells and rat cerebral cortical neurons to study Cd-induced oxidative stress. As shown in Figure 1A,B, Cd increased ROS generation in a dose- and time-dependent manner in SH-SY5Y cells, which was blocked by 2.5 mM antioxidant N-Acetyl-Cysteine (NAC) (Figure 1C). Furthermore, Cd significantly increased SOD2 expression in a time-dependent manner (Figure 1D). The SOD2 activity showed a peak after 6 h of 5 μM Cd exposure (Figure 1E). Moreover, in rat cerebral cortical neurons, ROS formation was induced by 5 μM Cd in a time-dependent manner (Figure 1F). These results indicate that Cd induces oxidative stress and activates antioxidant defense response in neuronal cells.

### 2.2. Cd Induces Nuclear Expression of FoxO3a in Neuronal Cells

**Figure 2:**
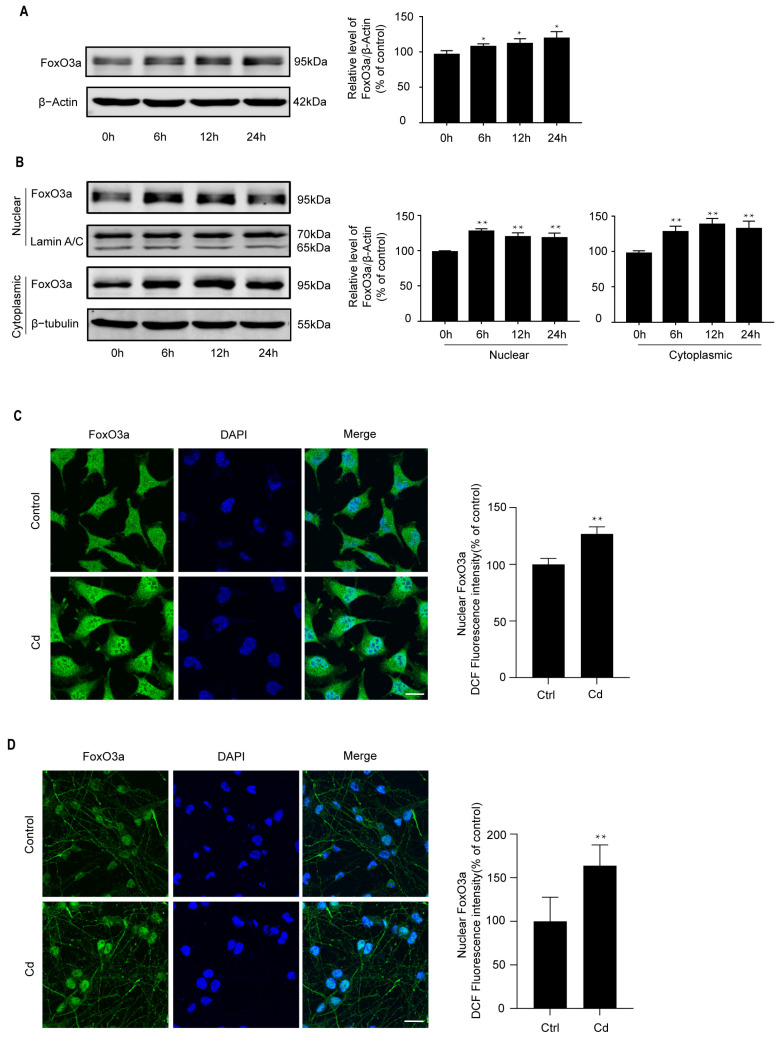
Cd induces the nuclear expression of FoxO3a in neuronal cells. SH-SY5Y cells were treated with 5 μM Cd for various time periods. (**A**) The cell lysates were subjected to Western Blot analysis to measure FoxO3a protein expression; (**B**) nuclear and cytoplasmic extracts were subjected to Western Blot analysis to measure FoxO3a expression. (**C**,**D**) SH-SY5Y cells and rat cerebral cortical neurons were exposed to 5 μM Cd for 6 h, and confocal laser scanning microscopy was performed to determine FoxO3a localization. FoxO3a is shown in green, and nuclei were counterstained with DAPI (blue). All results are representative of three independent experiments. The provided scale bar in the merged image represents 20 μm. * *p* < 0.05, ** *p* < 0.01 versus the control group.

FoxO3a can be activated by a variety of stresses and is closely related to oxidative stress resistance [28,29]. As shown in Figure 2A, Cd induced FoxO3a protein expression in a time-dependent manner. In response to 5 μM Cd treatment, FoxO3a protein expression increased at 6 h. To further determine whether Cd induces the nuclear expression of FoxO3a, Western Blot and confocal scanning laser microscopy were used to analyze FoxO3a expression. We found that Cd induces the nuclear expression of FoxO3a in SH-SY5Y cells (Figure 2B,C). Similar results were observed in Cd-treated rat cerebral cortical neurons at 6 h (Figure 2D). These findings suggest that low-dose Cd induces the nuclear expression of FoxO3a.

### 2.3. Knockdown of FoxO3a Increases Cd-Induced Oxidative Stress

**Figure 3:**
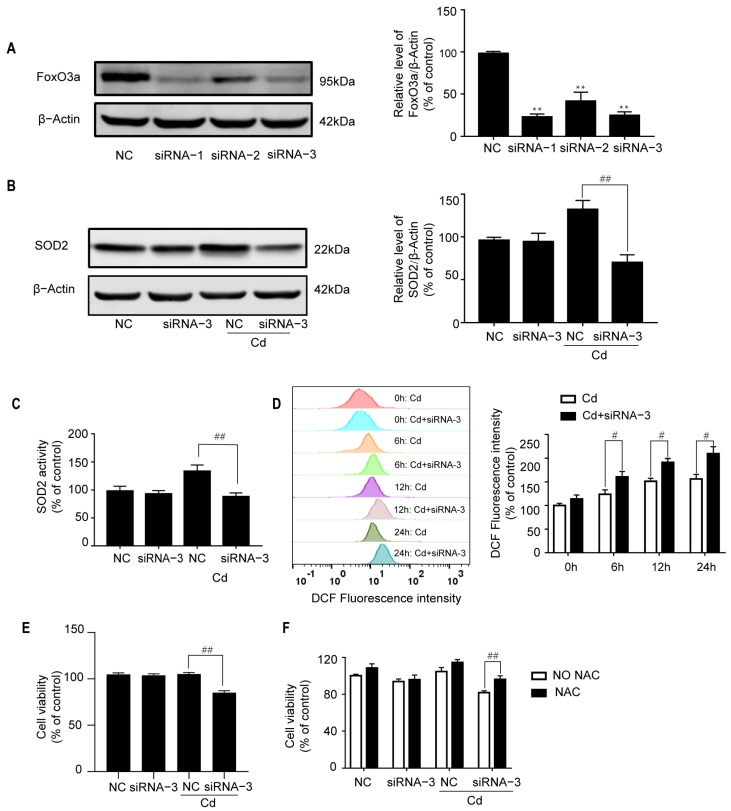
Knockdown of FoxO3a increases Cd-induced oxidative stress. (**A**) NC siRNA and siRNAs1-3 were transiently transfected into SH-SY5Y cells. Twenty-four hours after transfection, Western Blot analysis was performed to determine the FoxO3a protein level. Twenty-four hours after transfection with siRNA-3, SH-SY5Y cells were further exposed to 5 μM Cd for 6 h. SOD2 protein expression was analyzed by Western Blot (**B**), and SOD2 activity was measured by a CuZn/Mn-SOD Assay Kit (**C**). (**D**) Twenty-four hours after transfection, SH-SY5Y cells were treated with 5 μM Cd for various time periods, and ROS levels were analyzed by flow cytometry. (**E**) Twenty-four hours after transfection with siRNA-3, SH-SY5Y cells were treated with 5 μM Cd for 24 h, and subsequently, cell viability was analyzed by CCK8. (**F**) Twenty-four hours after transfection with siRNA-3, SH-SY5Y cells were exposed to 5 μM Cd for 24 h after preincubation with 2.5 mM NAC, and subsequently, cell viability was analyzed by CCK8. All results are representative of three independent experiments. ** *p* < 0.01 versus the control group; # *p* < 0.05, ## *p* < 0.01 versus the Cd group.

To ascertain the role of FoxO3a in Cd-induced oxidative stress, chemically synthesized siRNAs against *FoxO3a* were introduced into neuronal cells. Three siRNAs were screened, and Western Blot analysis showed FoxO3a protein levels in cells transfected with siRNA-1 or siRNA-3 were reduced to 20% of those found in cultures transfected with NC siRNA in SH-SY5Y cells (Figure 3A). As shown in Figure 3B,C, SOD2 expression and activity were greatly inhibited by siRNAi-3. Meanwhile, the level of intracellular ROS was greatly elevated (Figure 3D). Corresponding to these results, transfection with siRNA-3 significantly inhibited cell viability in Cd-exposed SH-SY5Y cells (Figure 3E), which is rescued by NAC (Figure 3F). These results suggest that FoxO3a plays an antioxidant role in Cd-treated neuronal cells.

### 2.4. p38 Regulates the Cd-Induced Nuclear Expression of FoxO3a

The Akt signaling pathway can inhibit nuclear expression of FoxO3a. To determine the association of FoxO3a expression and the Akt pathway, we measured the activity of Akt and phosphorylation of FoxO3a at its Akt phosphorylation sites (Ser253) [30]. Western Blot analysis showed the level of Akt phosphorylation (Ser473) and Akt remained at the base level after 6 h (Figure S1A). Moreover, the level of FoxO3a phosphorylation (Ser253) has no significant change (Figure S1B). These suggest that the Akt pathway does not play a major role in Cd-induced nuclear expression of FoxO3a.

**Figure 4:**
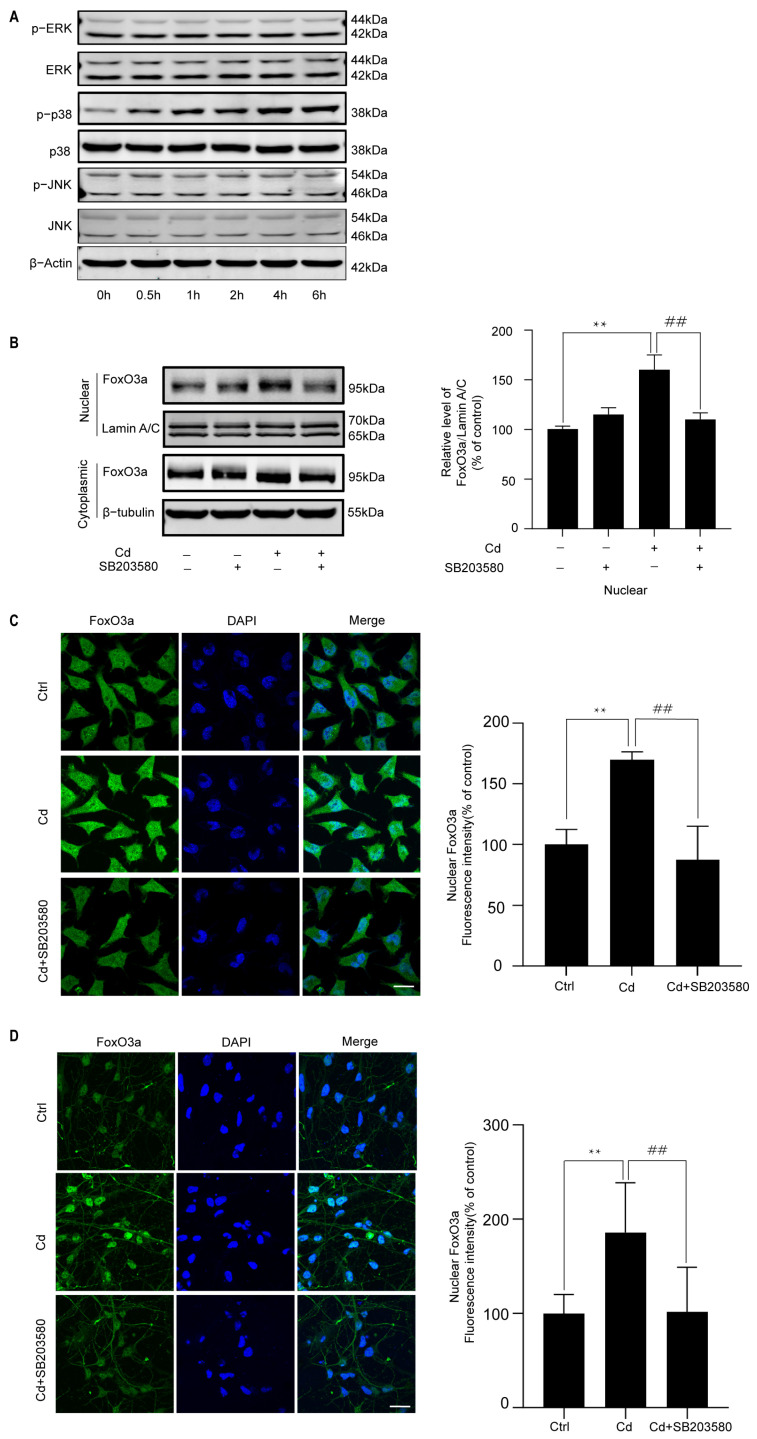

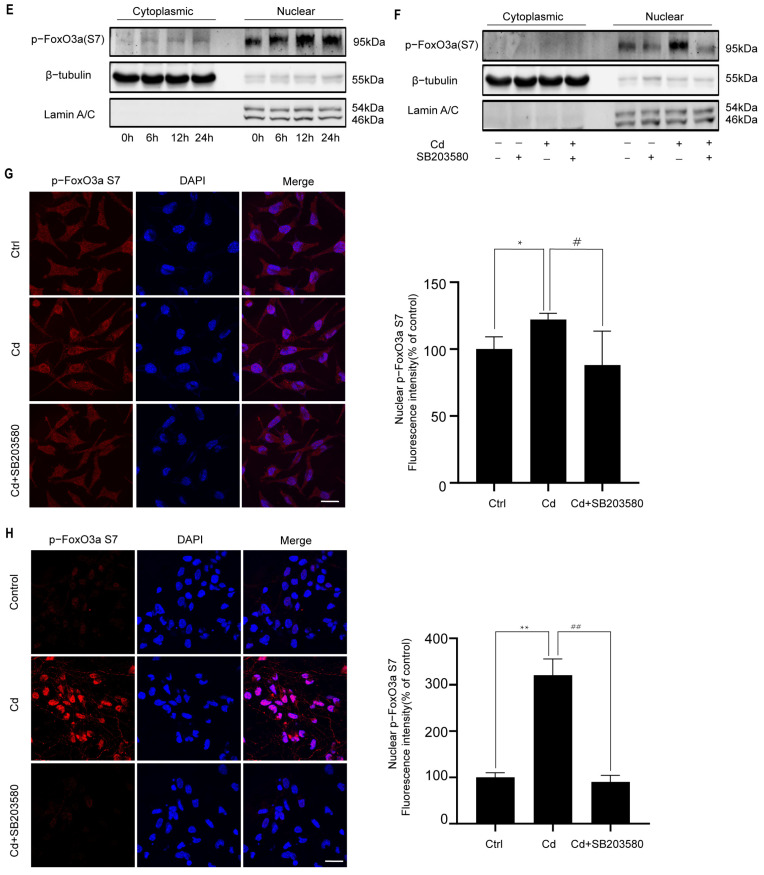
p38 regulates the Cd-induced nuclear expression of FoxO3a. (**A**) SH-SY5Y cells were treated with 5 μM Cd for various time periods, and phosphorylated and total MAPK proteins were detected by Western Blot analysis. (**B**) SH-SY5Y cells were treated with 5 μM Cd for 6 h after preincubating with 10 μM SB203580 for 1 h. Western Blot analysis was performed to determine FoxO3a expression. (**C**,**D**) SH-SY5Y cells and rat cerebral cortical neurons were exposed to 5 μM Cd for 6 h after preincubation with 10 μM SB203580 for 1 h. Confocal laser scanning microscopy was performed to determine FoxO3a expression. FoxO3a is shown in green, and nuclei were counterstained with DAPI (blue). (**E**) SH-SY5Y cells were treated with 5 μM Cd for various time periods. Western Blot analysis was performed to determine phosphorylation of FoxO3a-Ser7. (**F**–**H**) SH-SY5Y cells and rat cerebral cortical neurons were incubated with 5 μM Cd for 6 h after preincubation with 10 μM SB203580 for 1 h. Western Blot analysis and confocal laser scanning microscopy were performed to determine phosphorylation of FoxO3a-Ser7 expression. The provided scale bar in the merged image represents 20 μm. (**G**–**H**) p-FoxO3a is shown in red, and nuclei were counterstained with DAPI (blue). All results are representative of three independent experiments. * *p* < 0.05, ** *p* < 0.01 versus the control group; # *p* < 0.05, ## *p* < 0.01 versus the Cd group.

MAPKs have also been reported to regulate the intracellular expression of FoxO3a. Therefore, MAPK activation was investigated following Cd treatment. As shown in Figure 4A, Cd induced a time-dependent increase in p38 phosphorylation; however, phosphorylation of ERK and JNK was not increased until 6 h. To further assess the effect of p38 on the FoxO3a nuclear expression, neuronal cells were exposed to Cd after pretreatment with a p38 inhibitor, SB203580, for 1 h. We found that SB203580 suppressed Cd-induced nuclear expression of FoxO3a (Figure 4B–D). We further investigated the phosphorylation of FoxO3a at its p38 phosphorylation sites (Ser7). Cd significantly induced phosphorylation of FoxO3a (Ser7) in the nucleus (Figure 4E). Notably, pretreatment with a p38 inhibitor suppressed the effect (Figure 4F). We used confocal scanning laser microscopy to analyze FoxO3a phosphorylation (Ser7) expression. The results showed that SB203580 treatment decreased the expression of phosphorylated FoxO3a (Ser7) in the nucleus (Figure 4G). The nuclear expression of FoxO3a phosphorylation (Ser7) and the effect of SB203580 in Cd-treated rat primary cerebral cortical neurons were similar to those observed in the SH-SY5Y cells (Figure 4H). Taken together, these observations indicate that nuclear expression of FoxO3a induced by Cd is dependent on the p38 pathway.

### 2.5. The Effects of the p38/FoxO3a Pathway on the Cd-Induced Oxidative Stress

**Figure 5:**
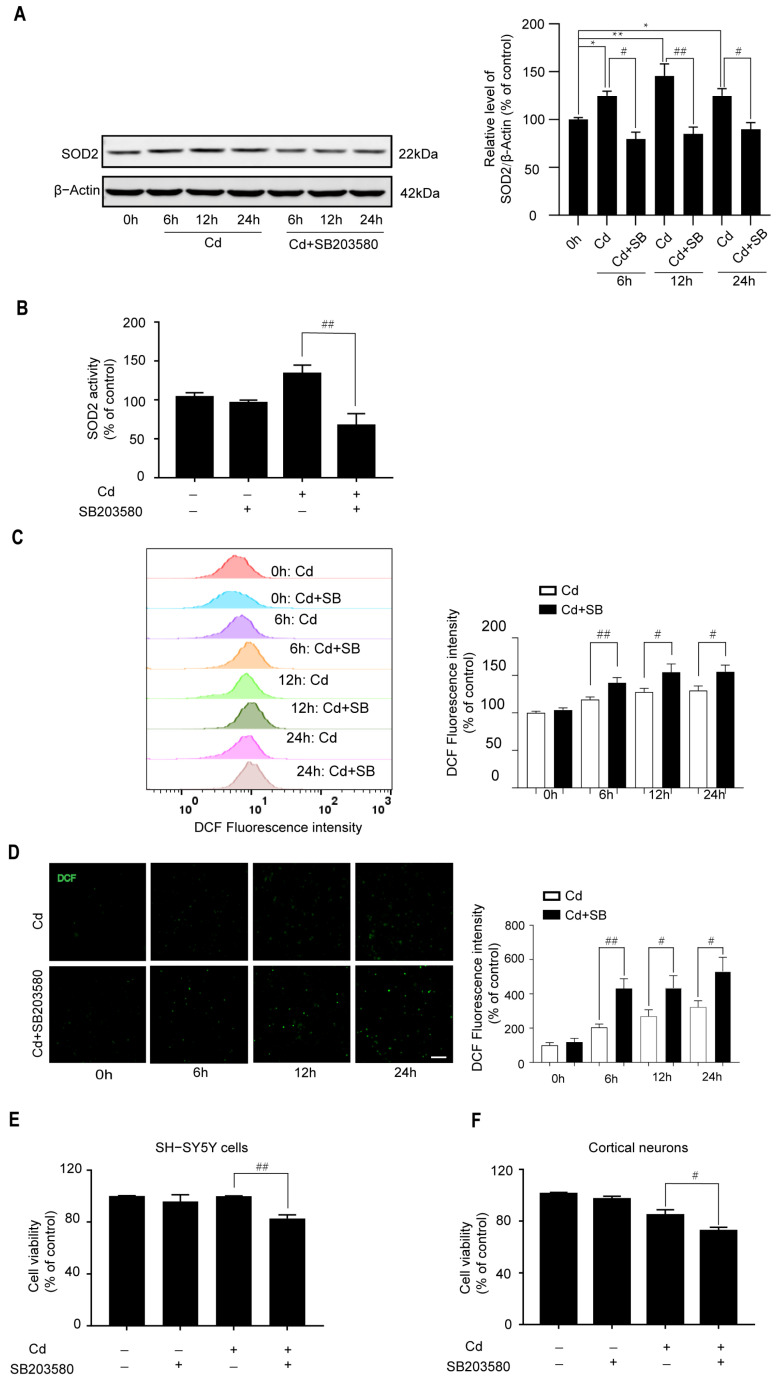
The effects of the p38/FoxO3a pathway on the Cd-induced oxidative stress. (**A**) SH-SY5Y cells were treated with 5 μM Cd for various time periods after being pretreated with 10 μM SB203580 for 1 h. Western Blot analysis was performed to determine SOD2 protein expression. (**B**) SH-SY5Y cells were treated with 5 μM Cd for 6 h after preincubating with 10 μM SB203580 for 1 h. A CuZn/Mn-SOD Assay Kit was used to measure SOD2 activity. SH-SY5Y cells and cerebral cortical neurons were preincubated with 10 μM SB203580 for 1 h, followed by treatment with 5 μM Cd for various time periods. ROS levels were analyzed by flow cytometry or fluorescence microscopy (**C**,**D**); cells were exposed to 5 μM Cd for 24 h after preincubation with SB203580, and subsequently, cell viability of SH-SY5Y cells and rat cerebral cortical neurons was analyzed by CCK8 (**E**,**F**). All results are representative of three independent experiments. The provided scale bar in the merged image represents 200 μm. * *p* < 0.05, ** *p* < 0.01 versus the control group; # *p* < 0.05, ## *p* < 0.01 versus the Cd group.

To determine the effects of the p38/FoxO3a pathway on the Cd-induced oxidative stress, neuronal cells were treated with SB203580. As shown in Figure 5A,B, pretreatment with SB203580 inhibited Cd-induced SOD2 expression and activity. Moreover, the level of intracellular ROS was greatly elevated by SB203580 (Figure 5C,D). More importantly, pretreatment with SB203580 displayed a certain damaging effect in Cd-treated neuronal cells (Figure 5E,F). Therefore, the p38/FoxO3a pathway participates in the resistance of Cd-induced oxidative stress.

## 3. Discussion

Cd in the diet and environment is a major source of Cd in humans, which may cause neurological disorders [31,32,33]. Cd can induce oxidative stress and probably trigger antioxidant responses that promote cell adaptation and survival [16,34]. In the current study, we found that Cd induced ROS generation in SH-SY5Y cells and cerebral cortical neurons. Meanwhile, the protein expression and activity of SOD2 are elevated with Cd exposure. These findings suggest that Cd induces oxidative stress and activates the antioxidant defense system in neurons. Moreover, we noted that SOD2 activity peaked at 6 h and subsequently returned towards baseline levels. It has been reported that SOD2 activity is positively regulated by SIRT3-mediated deacetylation [35]. Cd-induced SIRT3 reduction leads to the hyperacetylation and activity inhibition of SOD2 [36], which may account for the observed change in SOD2 activity under prolonged Cd exposure.

As an important transcription factor, FoxO3a is closely related to oxidative stress and cell death. It has been reported that the activation of FoxO3a can induce the expression of SOD2 [37]. However, the FoxO3a-SOD2 antioxidant pathway was inhibited by the Cd exposure in TCMK-1 cells [38]. Our study shows for the first time that Cd activates the FoxO3a-SOD2 antioxidant pathway, which attenuates Cd-induced ROS generation. This was confirmed by the following findings: (1) By combined use of Western Blot and confocal scanning laser microscopy, we found that Cd induces the nuclear expression of FoxO3a in SH-SY5Y cells and cerebral cortical neurons; (2) Knockdown of FoxO3a inhibits the protein expression and activity of SOD2; meanwhile, it increases intracellular ROS levels. Corresponding to the above results, the combination of Cd and *FoxO3a RNAi* exhibited significant cytotoxicity compared with the Cd group, which is partially rescued by NAC. These results suggest that FoxO3a plays an antioxidant role in Cd-treated neuronal cells. Moreover, FoxO1 is also associated with oxidative stress, and the relationship is complex. The activation of FoxO1 can promote oxidative stress [39,40,41]. It has been reported that FoxO1 increases Krüppel-like factor-5 expression, which in turn stimulates NADPH oxidase 4 expression and induces oxidative stress [39]. On the other hand, FoxO1 can also promote antioxidant responses [42]. The exact role and molecular mechanism of FoxO1 in Cd-induced oxidative stress warrant further investigation.

While SOD1 and glutathione also play roles in the cellular antioxidant defense, there is little evidence that their expression/synthesis is directly regulated by FoxO3a. Cd toxicity is widely recognized to target mitochondria. The primary mitochondrial antioxidant enzyme, SOD2, has been clearly reported as a direct transcriptional target of FoxO3a. This further reinforces the necessity of investigating the FoxO3a-SOD2 pathway. Recent research proposes that SOD2 can convert diffusion-restricted superoxide radicals into highly diffusible hydrogen peroxide [43]. Hydrogen peroxide, the diffusible secondary messenger, can not only transmit mitochondrial signals to the cytoplasm and/or nucleus but also regulate mitochondrial quality control. These novel functions and mechanisms of SOD2 deserve further investigation in the context of Cd toxicity.

The mechanism of FoxO3a nuclear expression induced by Cd remains unclear. As the most important negative regulator of FoxO3a, Akt is responsible for the phosphorylation of FoxO3a at the Ser253 site. This phosphorylation leads to the exclusion of FoxO3a from the nucleus [30]. In the current study, Cd had no effect on Akt phosphorylation (Ser473) and FoxO3a phosphorylation (Ser253). The results show that the Akt pathway does not play a major role in Cd-induced nuclear expression of FoxO3a. MAPKs are FoxO-activating kinases. In our previous studies, Cd induced the activation of MAPKs in vitro and in vivo, so we tested the activation of MAPKs [44]. We found that Cd induced p38 phosphorylation, while phosphorylation of ERK and JNK remained at basal levels. However, whether the activation of p38 is related to the FoxO3a nuclear expression remains unclear. p38 inhibitor suppressed Cd-induced nuclear expression of FoxO3a in SH-SY5Y cells and cerebral cortical neurons, indicating that p38 is involved in the nuclear expression of FoxO3a. To further ascertain the relationship between p38 and FoxO3a, we examined FoxO3a (Ser7), which can be specifically phosphorylated by p38. We found that FoxO3a (Ser7) increased in the nucleus after Cd exposure. Pretreatment with a p38 inhibitor significantly inhibited nuclear phosphorylation of FoxO3a (Ser7). Several studies have reported that p38 promotes nuclear expression of FoxO3a via phosphorylating its Ser7 site [45,46]. Consistent with the above studies, we found that p38 can phosphorylate the FoxO3a Ser7 site and promote its nuclear expression in Cd-treated neuronal cells. The nuclear expression of transcription factors is associated with nuclear import and nuclear export mechanisms [47,48,49,50]. Protein phosphorylation can affect nuclear transport by complex mechanisms [47,51]. The exact mechanism by which phosphorylation affects FoxO3a nuclear import or nuclear export requires further intensive research.

As an important kinase regulating oxidative stress, proliferation, and apoptosis, p38 has been shown to be activated in a wide range of stress. p38 works as a sensor of oxidative stress that influences the redox balance, ultimately determining the survival, proliferation, and senescence of cells [52]. To determine the role of p38 in Cd-induced oxidative stress, we pretreated cells with p38 inhibitors and found that SOD2 protein expression and activity were inhibited; meanwhile, intracellular ROS were accumulated. These indicate that p38 can inhibit Cd-induced oxidative stress. Although originally described as a mediator of apoptosis, p38 can exert a protective effect by regulating different downstream molecules under different stress states [53,54]. In our previous studies, p38 also plays a protective role in Cd-treated neuronal cells [44]. In the current study, we further demonstrate that p38 plays a protective role in Cd-induced oxidative stress via regulating nuclear expression of FoxO3a. However, it is unclear how phosphorylated FoxO3a regulates SOD2. Further studies are needed to address this issue.

In conclusion, Cd activates p38 to promote FoxO3a nuclear expression, leading to the upregulation of antioxidant enzyme SOD2 and the subsequent mitigation of oxidative stress. The present study is the first to explore the protective role of the p38-FoxO3a pathway and provide biological markers in Cd-induced oxidative stress.

## 4. Materials and Methods

### 4.1. Cell Culture

Human neuroblastoma SH-SY5Y cells were from the Type Culture Collection of the Chinese Academy of Sciences (CAS, Shanghai, China). SH-SY5Y cells were grown in RPMI 1640 media supplemented with 10% fetal bovine serum (both from Invitrogen, Carlsbad, CA, USA) and maintained in a humid incubator (37 °C, 5% CO_2_) and used at a low passage number (<13). For experiments, SH-SY5Y cells were seeded at a density of 2–4 × 10^4^ cells/cm^2^. Cd treatment was initiated after 1 day.

Primary rat cerebral cortical neurons were obtained from the cerebral cortex of one-day-old Sprague–Dawley rats, with a typical yield of approximately 3–4 × 10^6^ viable neurons per pup. One-day-old Sprague–Dawley rats were provided by the SPF animal facility of the Guangzhou Center for Disease Control and Prevention. The rats were euthanized by decapitation, after which the meninges were carefully removed. The cortical tissues were minced, digested in 0.25% trypsin (Gibco, Grand Island, NY, USA) for 15 min, and then dissociated into a single-cell suspension by mechanical trituration with a Pasteur pipette. The cells were plated at a density of 4.0–5.0 × 10^5^ cells/cm^2^ on poly-L-lysine (Sigma-Aldrich, St. Louis, MO, USA)-coated plastic coverslips. After 2 h in a humid incubator (37 °C, 5% CO_2_), the initial medium was replaced with Neurobasal Plus Medium supplemented with B27 (Invitrogen, Carlsbad, CA, USA), 2 mM glutamine (Gibco, Grand Island, NY, USA), and 100 μg/mL penicillin/streptomycin (Gibco, Grand Island, NY, USA). 24 h later, 10 μM cytosine arabinoside (Ara-C) (Sigma-Aldrich, St. Louis, MO, USA) was added to the culture medium to prevent glial cell contamination. Half of the medium was exchanged for fresh medium every 3 days. Cd treatment was initiated after the neurons were cultured for 7 days.

During the experiment, any cultures showing signs of contamination or poor cell health were excluded.

### 4.2. Reagents

Cadmium chloride was purchased from Sigma-Aldrich Inc. (St. Louis, MO, USA). SB203580 was purchased from Cell Signaling Technology Inc. (Danvers, MA, USA). SOD2 antibody was purchased from Proteintech Group Inc. (Chicago, IL, USA). All the other antibodies were purchased from Cell Signaling Technology Inc. (Danvers, MA, USA). SOD2 antibody (24127-1-AP) was purchased from Proteintech (Chicago, IL, USA). Anti-Foxo3a antibody (#2497), anti-Foxo3a (Ser7) antibody (#14724), anti-Foxo3a (Ser253) antibody (#9466), ERK (p44/42 MAPK) antibody (#9107), p-ERK (p-p44/42 MAPK) antibody (#13214), p38 antibody (#8690), p-p38 antibody (#9215), JNK antibody (#9252), p-JNK antibody (#4668), AKT antibody (#9272), and p-AKT (Ser473) antibody (#9271) were purchased from Cell Signaling Technology Inc. (Danvers, MA, USA). Goat anti-Rabbit IgG (H + L) Cross-Adsorbed Secondary Antibody A-11008 was purchased from Thermo Fisher Scientific (Waltham, MA, USA). Anti-rabbit IgG (#7074) and anti-mouse IgG (#7076) were purchased from Cell Signaling Technology Inc. (Danvers, MA, USA).

### 4.3. Cadmium Treatment

The experimental conditions for cadmium exposure were established through preliminary dose- and time-response studies in neuronal cells. We tested various concentrations of cadmium chloride (CdCl_2_; 0, 2.5, 5, 10, and 20 μM) and different exposure times (0, 6, 12, and 24 h). Based on initial assessments of ROS, 5 μM was identified as the lowest concentration and 6 h as the earliest time point to induce a significant response (Figure 1A,B) in SH-SY5Y cells. These conditions were confirmed to be effective in primary cortical neurons as well (Figure 1F). Therefore, a standard treatment of 5 μM CdCl_2_ for 6 h was used for all subsequent experiments, with the 0 μM group serving as the control. Individual culture dishes were randomly allocated to each treatment group. For each independent experiment, 3 experimental units (technical replicates/wells) were allocated to each treatment group.

### 4.4. Cell Viability

Cell viability was analyzed using the Cell Counting Kit-8 (CCK-8) (Dojindo Laboratories, Kumamoto, Japan) according to the manufacturer’s instructions. Briefly, neuronal cells were seeded into 96-well plates (SH-SY5Y cells:1 × 10^4^ cells/well, primary cortical neurons:1.2 × 10^5^ cells/well). After the cells were treated with cadmium as described in the experimental design section, 10 μL of CCK-8 solution was added to each well, and the plates were incubated for an additional 2 h at 37 °C in the dark. The absorbance was then measured at a wavelength of 450 nm using the Multiskan Mk3 microplate reader (Thermo Labsystems, Waltham, MA, USA). Cell viability was calculated as a percentage relative to the control group, using the formula: Viability (%) = [(OD_treatment − OD_blank)/(OD_control − OD_blank)] × 100%. Wells containing only culture medium and CCK-8 solution served as the blank.

### 4.5. Reactive Oxygen Species (ROS) Measurement

Intracellular ROS level was evaluated using CM-H2DCFDA (Molecular Probes; Molecular Probes, Invitrogen, Carlsbad, CA, USA), which is a stable nonfluorescent molecule and can passively diffuse into cells, where it is oxidized by ROS and becomes green fluorescent. Thus, the DCF fluorescence intensity is proportional to the amount of peroxides produced by the cells. Briefly, the harvested SH-SY5Y cells were incubated with DCFH-DA (10 μM) for 30 min in the dark at 37 °C. After treatment, cells were washed three times and resuspended in PBS. ROS generation was measured using a flow cytometer (FACSCalibur, BD Biosciences, San Jose, CA, USA). Cerebral cortical neurons were incubated with DCFH-DA, cells were washed three times with PBS, and then measured using a fluorescence microscope with a 10× objective.

### 4.6. Knockdown of FoxO3a

Knockdown of FoxO3a in SH-SY5Y cells was performed using siRNA transfection. Briefly, cells were seeded in 6-well plates (2.5 × 10^5^ cells/well) and cultured to 60–70% confluence. The cells were transiently transfected with either a specific siRNA targeting human *FoxO3a* (Thermo Fisher Scientific, Waltham, MA, USA, Catalog: 1299001, siRNA1:HSS177174; siRNA2: HSS177175; siRNA3: HSS177176) or a non-targeting negative control siRNA (Thermo Fisher Scientific, Waltham, MA, USA, Catalog: 12935-300) to a final concentration of 50 nM using Lipofectamine RNAiMAX Transfection Reagent (Invitrogen, Carlsbad, CA, USA, 13778150). The siRNA-lipid complexes were prepared in Opti-MEM medium (Gibco, Grand Island, NY, USA) according to the manufacturer’s protocol and incubated for 15 min at room temperature before being added to the cells. At 24 h post-transfection, the cells were treated with 5 μM CdCl_2_ for 6 h, and the knockdown efficiency was subsequently confirmed by Western Blot analysis.

### 4.7. Western Blot Analysis

Proteins were separated by sodium dodecyl sulfate-polyacrylamide gel electrophoresis (SDS-PAGE) and transferred to PVDF membranes (Immobilon-P, Millipore, Burlington, MA, USA). Membranes were blocked for 1 h at room temperature and then incubated overnight with the primary antibodies. The membranes were incubated with secondary antibody for 1 h at room temperature, and the signal was detected by the Odyssey Infrared Imaging System (LI-COR Biosciences, Lincoln, NE, USA).

### 4.8. Immunofluorescence

The cells were fixed in 4% paraformaldehyde (Sigma-Aldrich, St. Louis, MO, USA) for 15 min at room temperature to analyze the expression of FoxO3a in the nucleus. The cells were permeabilized with frozen methanol for 10 min at −20 °C and then blocked in 5% BSA (Sigma-Aldrich, St. Louis, MO, USA) for 30 min. The cells were incubated with primary antibody (1:100) in 5% BSA overnight at 4 °C, and then incubated with an Alexa Fluor 488 or 594 antibody (Invitrogen, Carlsbad, CA, USA) at a 1:500 dilution for 1 h at room temperature. The cells were incubated with DAPI (Cell Apoptosis DAPI Detection Kit, KGA215, KeyGEN BioTECH, Nanjing, China) according to the manufacturer’s instructions. An Olympus FluoView™ FV1000 confocal laser scanning microscope with a 100× objective was used to record the resultant images.

### 4.9. Measurement of SOD2 Enzyme Activity

SOD2 enzymatic activity was assayed using a CuZn/Mn-SOD Assay Kit with WST-8 (Beyotime, S0103, Nanjing, China) following the manufacturer’s instructions. To ensure the specific measurement of mitochondrial SOD2 activity and differentiate it from cytosolic Cu/Zn-SOD (SOD1), the assay was conducted in the presence of specific inhibitors that block the activity of SOD1, as per the kit’s protocol. This step allows for the isolated detection of SOD2 activity. The absorption at 450 nm was measured using a Multiskan Mk3 microplate reader (Thermo Labsystems, Waltham, MA, USA).

### 4.10. Statistical Analysis

All data in the figures are presented as the mean ± standard error of the mean (SEM) of at least three independent experiments performed in duplicate. Statistical analyses were performed using SPSS software (version 26.0, IBM Corp., Armonk, NY, USA). A Student’s *t*-test was used to determine the statistical significance of the difference in values between two groups. One-way ANOVA was used for statistical analysis of differences in values among multiple groups. *p* < 0.05 was considered significant.

## Data Availability

All data that support the findings of this study are available from the corresponding author upon reasonable request.

## Supplementary Materials

The following supporting information can be downloaded at: https://www.mdpi.com/article/10.3390/ijms262210919/s1.
