# Supplementary material for: p38 Regulates FoxO3a-Mediated SOD2 Expression to Prevent Cd-Induced Oxidative Stress in Neuronal Cells
Source: Int J Mol Sci. 2025 Nov 12;26(22):10919. doi: 10.3390/ijms262210919 (PMC12652648; doi:10.3390/ijms262210919)
Supplement: Supplementary file 1 [file ijms-26-10919-s001.zip › ijms-3963361-supplementary.pdf]

International Journal of *Molecular Sciences*

Article

p38 Regulates FoxO3a-Mediated SOD2 Expression to Prevent Cd-Induced Oxidative Stress in Neuronal Cells

Tianji Lin [1,2], Shijuan Ruan [1,3], Xinyu Liu [1], Fangfei Li [1], Hangqian Zhang [1], Fei Zou [1,*] and Bin Wang [1,*]

1 Department of Occupational Health and Occupational Medicine, School of Public Health, Southern Medical University, Guangzhou 510515, China; lintianji@126.com (T.L.); ruansj2025@163.com (S.R.); 13060604992@163.com (X.L.); 13172396244@163.com (F.L.); 15257956196@163.com (H.Z.)
2 Guangzhou Center for Disease Control and Prevention (Guangzhou Health Supervision Institute), Guangzhou 511400, China
3 Sichuan Center for Disease Control and Prevention, Chengdu 610041, China
* Correspondence: zfei@smu.edu.cn (F.Z.); wenwunj@smu.edu.cn (B.W.)

Supplementary Materials

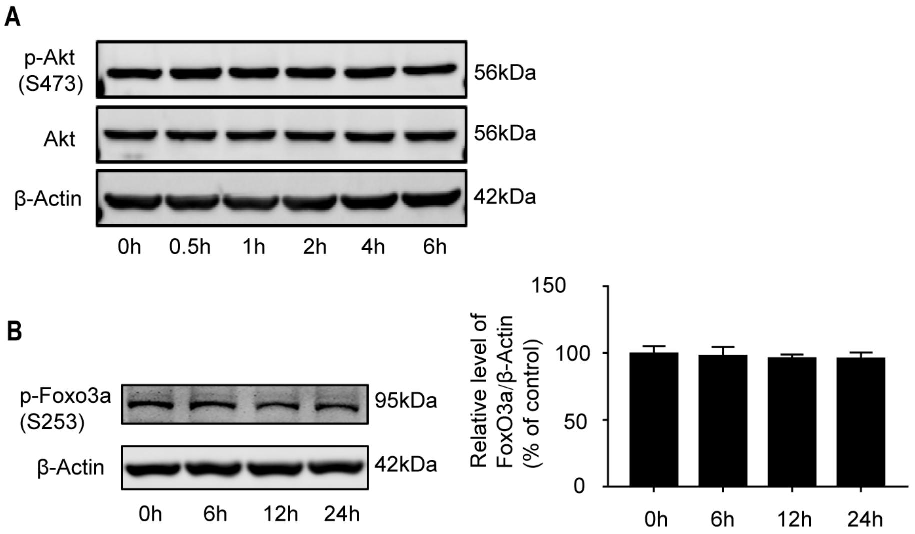

Figure S1. Akt is not involved in the Cd-induced nuclear expression of FoxO3a. SH-SY5Y cells were treated with 5μM Cd for indicated times. Western blot analysis was performed to determine phosphorylated and total Akt protein (A), and phosphorylation of FoxO3a (Ser253) (B). All results are representative of three independent experiments(n=3)..
